# Achieving High Excipient Efficiency with Elastic Thermoplastic Polyurethane by Ultrasound Assisted Direct Compression

**DOI:** 10.3390/pharmaceutics11040157

**Published:** 2019-04-02

**Authors:** Eduardo Galdón, Marta Casas, Isidoro Caraballo

**Affiliations:** Departamento de Farmacia y Tecnología Farmacéutica. Facultad de Farmacia, Universidad de Sevilla, 41012 Seville, Spain; egaldoncabrera@gmail.com (E.G.); caraballo@us.es (I.C.)

**Keywords:** ultrasound assisted direct compression, polyurethanes, prolonged-release tablets, percolation theory, porosity, fractal dimension

## Abstract

Ultrasound assisted compression (USAC) is a manufacturing technique which applies thermal and mechanical energy to the powder bed, producing tablets with improved characteristics compared to the direct compression process. This technology is ideal for thermoplastic materials, as polyurethanes, whose particles usually undergo a sintering process. Thermoplastic polyurethanes are widely used in sustained drug release systems but rarely seen in tablets due to their elastic properties. The aim of this work is to investigate the ability of USAC to manufacture sustained release matrix tablets based on elastic thermoplastic polyurethanes (TPU), overcoming the limitations of direct compression. The technological and biopharmaceutical characteristics of the TPU matrices have been evaluated, with special focus on the porous structure due to the implications on drug release. For the first time, USAC has been successfully employed for manufacturing elastic thermoplastic polyurethanes-based matrices. TPU tablets show an inert character with a sustained drug release governed by a diffusional mechanism. Initial porosity of matrices was similar in all batches studied, with no influence of drug particle size, and a fractal nature of the pore network has been observed. SEM microphotographs show the continuum medium created by the sintering of the polymer, responsible for the high excipient efficiency.

## 1. Introduction

Nowadays, tablets remain as the most common dosage form in the pharmaceutical market. This proportion has even increased thanks to the generic drugs since tableting generally results in a lower cost of development as well as a higher industrial production capacity [1]. However, in the majority of cases, either wet or dry granulation has to precede the compaction step to obtain suitable properties for industrial processing and to achieve the targeted properties of the finished drug product.

Ultrasound-assisted compression (USAC) is a manufacturing technology which combines a conventional compression process with ultrasound irradiation. Ultrasound energy causes mechanical and thermal effects which lead to heating and sintering of materials, improving the compression process [2]. Thus, ultrasounds increase the interparticle bonds resulting in stronger and less porous tablets at lower pressures as compared to conventional tableting [3,4,5]. Moreover, the use of intermediate processes as granulation is also avoided with USAC.

This technology has been successfully employed for different applications as the amorphization of active pharmaceutical ingredients, production of solid dispersions to enhance the bioavailability of poorly soluble drugs and formulation of sustained drug release forms [3,6,7,8,9,10]. In the case of prolonged release systems, USAC appears as a promising technique, decreasing the cost and the quantity of excipient needed for the control of the drug release [8], which usually constitutes an important drawback in the formulation of sustained-release systems.

This process is particularly suitable when the excipient exhibits a thermoplastic deformation, allowing the formation of a framework that holds the non-thermoplastic particles together. In this way, the polymer will form an inert skeleton avoiding the disintegration of the matrix, surrounding more efficiently the drug, and consequently controlling the drug release with a low quantity of excipient.

Thermoplastic polyurethanes (TPU) have stood out as a very interesting group of excipients for controlled release dosage forms, being successfully used in vaginal rings, stents, coatings, and implants due to favorable properties as its inert, non-ionic, and water-insoluble character. They also exhibit a high tensile strength, crack resistance, inherent lubricity, and highly elastomeric character. Recently, TPU have been researched for the manufacturing of oral sustained drug release forms obtained by hot-processing techniques, such as hot melt extrusion or injection molding [11,12]. However, the manufacture of TPU tablets by direct compression has been very limited due to their poor plastic/elastic balance at room temperature [13]. 

Due to the water insoluble character of TPU, the porosity of the inert matrices obtained plays an important role in their performance as it directly affects the liquid uptake rate and influences the drug release kinetics. Normally, the pore structure is complex and randomly distributed with different sizes, shapes, and orientations, which makes it difficult to accurately describe it [14]. Therefore, different techniques have been employed to determine macroscopic and microscopic pore parameters, since its analysis becomes necessary for the deeper knowledge of these systems.

The aim of this work is to investigate the ability of USAC to overcome the undesirable elastic properties of thermoplastic polymers, allowing one to obtain tablets by direct compression. An additional objective is the study of the properties of the obtained tablets.

For this purpose, we employed the elastic thermoplastic polyurethane (TPU) Tecoflex^TM^ EG-72D as the matrix forming excipient in tablets obtained by USAC. The main properties of the obtained high drug content TPU matrices have been evaluated, including the study of their porous system through fractal dimension and the influence of drug particle size.

## 2. Materials and Methods 

### 2.1. Materials

Anhydrous theophylline (batch151209-P-1, Acofarma, Barcelona, Spain) was used as model drug. Medical grade elastomer thermoplastic polyurethane Tecoflex^TM^ EG-72D (TPU) was used as matrix forming excipient, which was kindly supplied by Lubrizol Advanced Materials Spain S.L. (Bacelona, Spain). The TPU chemical structure consists of polytetrahydrofuran (soft segment) and hydrogenated methylene diphenyl diisocyanate (hard segment) at a molar ratio of 3.5. The molecular weight is 59,000 g/mol and its melting point is 53° [11]. TPU ultimate tensile strength and elongation at break are 55.8 MPa and 310%, respectively.

### 2.2. Methods

#### 2.2.1. Blends Preparation 

Tecoflex^TM^ EG-72D pellets were frozen in liquid nitrogen and subsequently milled with a Restch ZM 200 equipment (Haan, Germany), using a 1.0 mm output sieve. Three fractions of drug particle size (<90 μm, 90–150 μm and 150–355 μm) were obtained by sieving in order to evaluate the influence of this factor on the technological and biopharmaceutical behavior of the systems. 

Blends of theophylline and polymer powder (70/30 *w*/*w* proportion) have been mixed during 15 min (Turbula mixer, Willy A. Bachofen, Basel, Switzerland). The optimum blend time was calculated based on the drug content of 5 representative samples at different times tested. The quantity of drug was determined by UV–Vis spectrophotometry (Agilent 8453 (California, CA, USA)) at 272 nm [15]. Variation coefficient values lower than 5% were considered as appropriate.

#### 2.2.2. Preparation of TPU Tablets by Direct Compression

An eccentric tableting machine (Bonals A-300, Barcelona, Spain) was used to compact 300 mg of blends, using manual feeding and applying the maximum compression force accepted by the formulation.

#### 2.2.3. Preparation of TPU Tablets by Ultrasound-Assisted Direct Compression

Tablets of 300 mg weight for each lot were obtained using an ultrasound-assisted tableting machine (Tecnea Engineering, Casale Monferrato, Italy). An ultrasonic energy of 650 J was applied to the mixture at 20 kHz frequency. Flat cylindrical punches of 11 mm were employed. The parameters established for the proper compression were: compression pressure 0.3 MPa, compaction time 6 s, cool time 9 s and detach time 0.5 s.

#### 2.2.4. Physical Characterization of TPU Tablets 

The weight (EP214, Ohaus Corporation, Parsippany, NJ, USA), thickness and diameter (VWR International, Leuven, Belgium) of the TPU tablets were determined as the mean of 10 tablets for each lot.

#### 2.2.5. Dissolution Testing of TPU Tablets and Modelling 

Drug release studies were carried out using a USP Apparatus II Sotax AT7 smart (Allschwil, Switzerland) with 900 mL of deionized water at 37 ± 0.5 °C and 50 rpm. Samples were withdrawn at specific interval times and filtered through 0.45 mm filters (Millipore Ltd., Cork, Ireland). The percentage of drug released was measured in a UV–Vis spectrophotometer Agilent 8453 (California, CA, USA) at 272 nm [15]. The assay was performed in triplicate and sink conditions were met throughout the dissolution test. 

Drug release data (*M*_t_/*M*_∞_ ≤ 0.6) were analyzed according to Higuchi (1963) [16], Korsmeyer et al. (1983) [17] and Peppas and Sahlin (1989) [18] Equations (1)–(3):

(1) MtM∞=kt12,

(2) $$\frac{M_{t}}{M_{\infty }}=k_{k}t^{n},$$

(3) $$\frac{M_{t}}{M_{\infty }}=k_{d}t^{m}+k_{r}t^{2m},$$

where *M*_t_/*M*_∞_ is the drug released fraction at time t (the drug loading was considered as *M*_∞_), *k* is the Higuchi’s release rate constant, *k*_k_ is the Korsmeyer’s kinetic constant, *t* the release time, *n* the release exponent that depends on the release mechanism and the shape of the matrix tested [19], *k*_d_ and *k*_r_ are, respectively, the diffusion and relaxation rate constants, and finally *m* which is the purely Fickian diffusion exponent for a device of any geometrical shape which exhibits controlled release.

#### 2.2.6. Mercury Porosimetry Measurements

Mercury porosimetry runs were undertaken using an Autopore IV 9510 (Micromeritics, Madrid, Spain) porosimeter with a 3 cm^3^ penetrometer. An adequate number of tablets per tested formulation was used in order to obtain a stem volume between 25% and 90% of the penetrometer capacity. Working pressures covered the range 0.1–60,000 psi. Total porosity and pore size distribution of tablets were determined before and after the drug release study in duplicate and for each tested batch.

In the case of leached tablets, the porosity results have been normalized by subtracting the initial mercury intrusion, in order to perform a clearer comparison of the structure of the pore network inside these systems.

#### 2.2.7. Measurement of Fractal Dimension 

Volume fractal dimension *D*_v_ of TPU matrices has been estimated according to the idealized model of the Menger sponge [20,21]. This model correlates the relative density of the system with the pore diameter filled with mercury at a certain intrusion pressure through the following equation (Equation (4)):

(4) $$\log\rho_{r}=\left(3-D_{v}\right)logd+c,$$

where the relative density *ρ*_r_ is obtained by the pore volume fraction filled with mercury intrusion pressure *ρ*_r_ = 1 − *ε* and *c* is a constant.

Volume fractal dimension can be calculated by the slope of the straight line obtained by plotting the relative density of the system *ρ*_r_ as a function of diameter in a double logarithmic plot.

#### 2.2.8. Scanning Electron Microscopy (SEM) 

The surface of TPU matrices were evaluated at the Microscopy Service of the CITIUS in the University of Seville by using Scanning Electron Microscopy (SEM) with a FEI TENEO electronic microscope (FEI Company, Hillsboro, OR, USA), operating at 5 kV. Tablets were coated with a 10 nm thin Pt layer with Leica EM SCD500 high vacuum sputter coater.

#### 2.2.9. Estimation of the Excipient Efficiency

The Excipient Efficiency is a parameter to quantify the ability of an excipient to control the drug release from a pharmaceutical formulation [15]. This parameter has been calculated for TPU according to the following equation (Equation (5)):

(5) $$E=\frac{\varepsilon }{b}\;\frac{1}{\left(1.43-0.00244d\right)}\;\frac{1}{\left(1.963-0.246lnC_{s}\right)},$$

where *ε* is the total porosity of the matrices, *b* is the Higuchi’s release rate constant, *d* is the mean particle size of excipient (µm) and *C*_s_ is the drug solubility in mg/mL. 

## 3. Results and Discussion

Tablets of TPU and theophylline were successfully obtained by USAC for all batches. Conversely, when the same blends of TPU and theophylline were processed through a standard eccentric tableting machine, the obtained tablets do not show suitable technological characteristics, mainly due to their elastic properties leading to a crumbling of tablets during the relaxation step (in less than 24 h). Therefore, all the following sections, devoted to the characterization of the obtained tablets, are referred to the USAC tablets.

### 3.1. Characterization of TPU Tablets Obtained by USAC

Physical characteristics of tablets were consistent with the established compression conditions, obtaining an average weight, diameter, and thickness of 297.3 ± 1.0 mg, 11.247 ± 0.000 mm, and 2.856 ± 0.034 mm, respectively. Tablet hardness or tensile strength of tablets could not be measured due to the elastic nature of the system. Tablets were deformed instead of broken when they were subjected to the crushing strength tester.

### 3.2. Dissolution Testing of TPU Tablets and Modelling 

**Figure 1:**
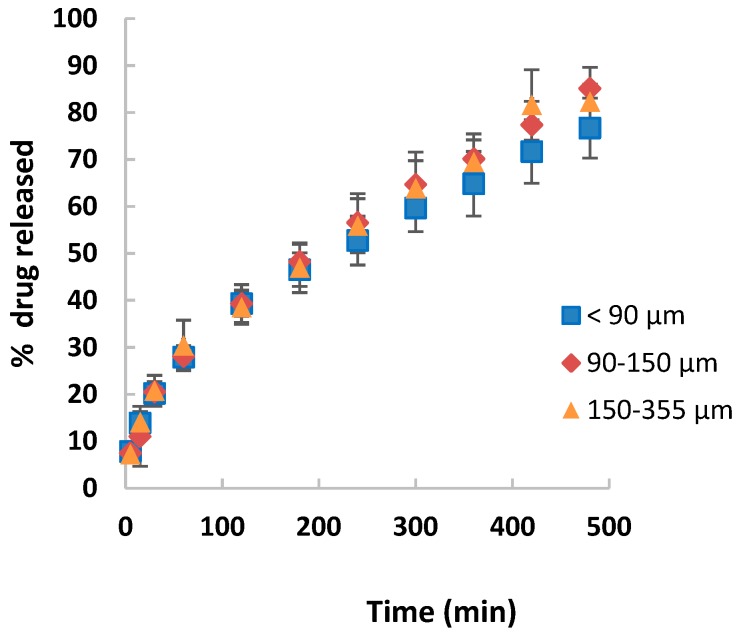
Drug release profiles of thermoplastic polyurethanes (TPU) tablets with different drug particle size.

Drug release profiles of TPU tablets are shown in Figure 1. Matrices made with different particle size of drug display similar biopharmaceutical behavior, achieving a prolonged theophylline release during more than 8 h. The integrity of tablets was maintained in all cases at the end of the study. 

On the basis of percolation theory, pharmaceutical systems can be described as randomly distributed materials where geometrical phase transitions can occur [22,23]. When a component reaches its percolation threshold, it starts to extend over the whole sample, having much greater influence on the properties of the system. 

In our case, as the excipient undergoes thermoplastic deformation, the continuum percolation model can be used to predict the changes in the system. This model considers a continuum distribution function of the components and the percolation threshold of a substance is situated at approximately 16% *v*/*v* of occupation probability [2]. According to the densities of the components, TPU constitutes 37.5% *v*/*v*, being therefore above its percolation threshold. This is consistent with the inert matrix formed and the controlled drug release obtained. With respect to the drug, being at a proportion of 62.5% *v*/*v* is also clearly above its percolation threshold. That ensures the complete release of the drug dose. 

Drug release data have been analyzed according to different kinetic models: Higuchi, Korsmeyer and Peppas and Sahlin (Table 1). The good determination coefficients for the diffusional model (Higuchi), the Korsmeyer *n* values close to 0.5, and the predominance of *k*_d_ over *k*_r_ in the Peppas and Sahlin equation reveal a drug release mechanism predominantly controlled by drug diffusion.

### 3.3. Mercury Porosimetry Measurements

**Figure 2:**
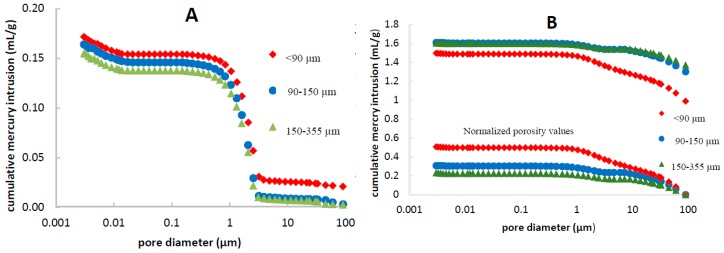
Cumulative mercury intrusion of USAC tablets with different drug particle sizes, before (**A**) and after drug release study (**B**), with normalized porosity values—without initial mercury intrusion—represented at the bottom of the image.

As TPU tablets are inert matrix systems, the knowledge of total porosity—the sum of the initial pores plus the pores that appear once the drug is dissolved—is critical to ensure the complete drug leaching and plays an important role in the release kinetics. TPU tablets showed initial porosity values between 17.1%–19.8% with a median pore diameter around 2 μm (Table 2). Figure 2A shows the cumulative mercury intruded by the matrices at different pressures, where no difference of porosity has been found as a function of drug particle size. Porosity of matrices after dissolution testing (total porosity) was measured since the integrity of tablets was maintained in all cases at the end of the assay. 

In the case of leached tablets, the obtained results showed different porosity patterns depending on particle size, as shown in Figure 2B. Matrices containing larger drug particle size have higher mercury intrusion at atmospheric pressure, due to the presence of higher pores left by the coarser drug particles after leaching [24]. Normalized porosity results, in which the initial mercury intrusion value has been subtracted, have been represented in the same Figure 2B in order to study the pore network. A more extensive pore network can be observed for the case of matrices with smaller particle size. 

As indicated in previous sections, the influence of drug particle size on the release profiles was sparingly significant. This can be due to the existence of two opposite influences. On one hand, the wider pores left by the coarser particles favor a faster diffusion through a reduction of the thickness of the diffusion layer. On the other, the matrices containing finer particles are expected to have a lower drug percolation threshold [25]. Therefore, they have a higher distance to the drug percolation threshold and consequently, they are expected to contain a more extended drug network than the coarser particles. The first one of these opposite phenomena is reflected in the absolute values of mercury intrusion, and the second one in the behavior of the normalized porosity results (Figure 2B).

**Figure 3:**
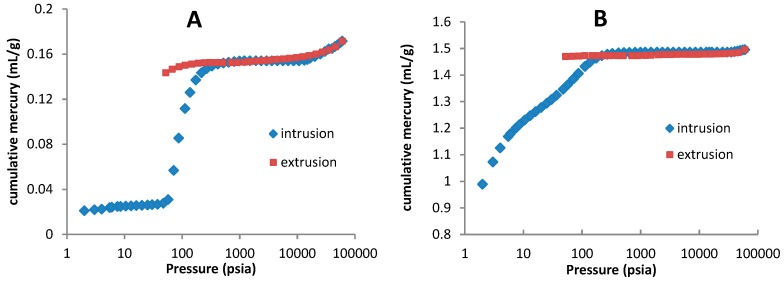
Mercury intrusion and extrusion curves from TPU tablets (**A**) and leached tablets (**B**).

The elastic nature of the TPU was evidenced since the extrusion curves follow the same plotted path as the intrusion curves (Figure 3). In contradistinction to non-elastic materials that show a typical pore retention hysteresis at extrusion process, TPU tablets seems to return to its original shape after being elastically compressed at the highest pressures, expelling the mercury [14,26].

### 3.4. Measurement of Fractal Dimension 

**Figure 4:**
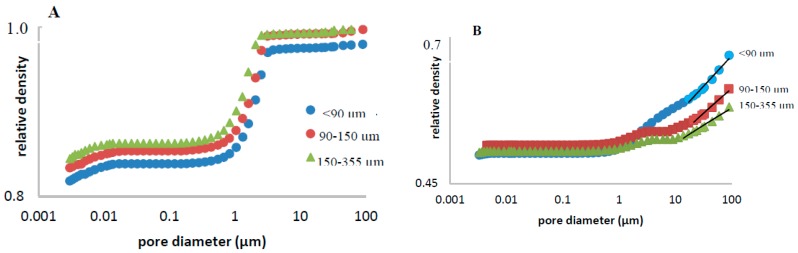
Determination of the volume fractal dimension of TPU matrices containing three different size fractions of theophylline, before (**A**) and after drug release study (**B**).

Fractal analysis, based on the concept of self-similarity, contributes to the knowledge of these porous systems, assigning them a fractional number which provides information about their structural complexity [27,28,29]. Volume fractal dimensions have been calculated for all matrices according to equation 4. Table 2 shows the obtained *D*_v_ values, which are in the range of 2.88–2.95, similar to previously reported values for sustained release matrices [20]. Higher *D*_v_ values have been obtained for matrices after drug dissolution test. In our study, self-similarity, which is characterized by a constant fractal dimension, was restricted to relatively narrow pore ranges: around 0.5 to 5, and around 10–100 micrometers for initial pores and pores formed after drug leaching, respectively (see Figure 4). Comparing the values for the different theophylline particle sizes studied, higher fractal nature was found when decreasing drug particle size, as observed by other researchers [20].

### 3.5. Scanning Electron Microscopy (SEM) 

**Figure 5:**
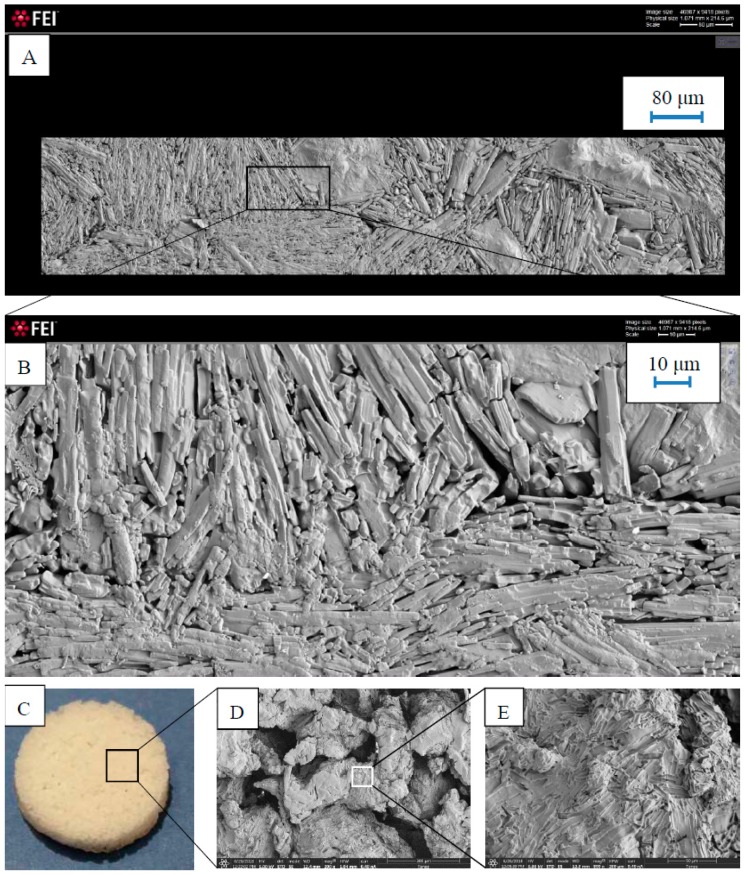
SEM images of USAC tablets before (**A**,**B**) and after drug dissolution (**D**,**E**). (**C**) is a digital photograph of the matrix after dissolution testing. Microphotograph 5A was obtained by Microsoft HD View 3.3 software. Magnifications of microphotographs 5B, 5D, and 5E are 5920×, 200×, and 800×, respectively.

Microphotographs obtained by SEM contributed to analyze the behavior of the polymer in the matrices. Images were taken before and after drug release studies, showing the continuum medium created by the thermoplastic excipient with only 30% weight fraction (Figure 5). This continuum medium of the polymer has been formed thanks to the ultrasounds applied by the upper punch-sonotrode which lead to the movement of the powder particles increasing the friction and collisions between them and rising the temperature of the system [2]. So, the boundaries between particles become indistinguishable causing the sintering of the system. Some researchers have also confirmed the sintering process obtained with USAC by SEM [5,6,7,30]. It can be highlighted that acicular drug particles tend to be distributed in parallel thus offering the lowest porosity previously measured, and how TPU works binding these particles (Figure 5A,B). SEM images of matrices after drug release study show the inert skeleton of TPU on which the fingerprints of theophylline particles are patent (Figure 5D,E). 

### 3.6. Estimation of the Excipient Efficiency

Considering the current regulatory framework, which encourages the application of the “Quality by Design” approach in pharmaceutical product development, we have estimated the parameter Excipient Efficiency (EE), in order to quantify the capability of Tecoflex^TM^ EG-72D for controlling the drug release. The mean EE value for the studied elastic thermoplastic polyurethane is 13.41 min^½^·mg^−1^·mL, according to the total porosity and the Higuchi’s release constant from the inert matrices. The obtained value is higher than those previously reported for inert matrix forming excipients as Ethocel^®^ (9.54–9.89) or Eudragit^®^ (5.59) [15]. This fact may be due to the compression process since the sintering process caused by USAC has been reported to increase the EE [7]. 

The capability of TPU for controlling drug release has been estimated for theophylline, which is slightly soluble in water. Obviously, in the case of lipophilic drugs, much lower release rates are expected. Taking into account the biocompatibility of this polymer, it would be possible to develop drug loaded implant devices using this technology.

## 4. Conclusions

Ultrasound-assisted compression (USAC) has demonstrated its ability to compress elastic materials, overcoming the traditional limitations of these materials for compression. For the first time, an elastic thermoplastic polymer has been successfully compressed by USAC, resulting in inert matrices with a semi-continuum excipient structure formed by the sintering process of these elastic TPU particles. This technology allows manufacturing matrices with low quantity of polymer (30%) showing high excipient efficiency. A fractal nature has been found in the pore structure. Porosity analysis has contributed to the understanding of the drug release behavior of TPU matrices, confirming the robustness of the obtained USAC matrices for varying drug particle size fractions. 

## Figures and Tables

**Table 1:** Main kinetic parameters from TPU tablets.

TPU Tablets with Different Drug Particle Size (μm)	Higuchi		Korsmeyer		Peppas & Sahlin		
	*b* (min^−0.5^)	*r* ^2^	*n*	*r* ^2^	*k*_d_ (min^−0.5^)	*k*_r_ (min^−1^)	*r* ^2^
<90	0.0339	0.9986	0.4915	0.9988	0.0387	−2.10^−4^	0.9994
90–150	0.0373	0.9966	0.5366	0.9896	0.0361	9.10^−5^	0.9974
150–355	0.0354	0.9947	0.513	0.9938	0.0361	−2.10^−6^	0.9957

*b*, Higuchi kinetic constant; *n*, release exponent; *k*_d_, Peppas diffusion kinetic constant; *k*_r_, Peppas relaxation kinetic constant; *r*^2^, determination coefficient.

**Table 2:** Porosity and fractal dimension *D*_v_ of TPU matrices obtained by ultrasound assisted compression (USAC), containing three different fractions of theophylline.

**Drug Fraction**	**TPU Matrices**	**Porosity** **(%)**	**Median Pore Diameter (μm)**	***D*_v_** **(and range in μm)**
	<90 μm	17.9 ± 0.7	2.1	2.883 (3.2–1.1)
	90–150 μm	19.8 ± 2.9	1.8	2.882 (2.5–1.1)
	150–355 μm	17.1 ± 1.9	1.7	2.899 (2.5–0.8)
**Drug Fraction**	**TPU Matrices** **after Dissolution Testing**			
	<90 μm	59.1 ± 0.4	33.7	2.9203 (17.2–90.6)
	90–150 μm	58.6 ± 1.8	41.1	2.9344 (21.3–90.3)
	150–355 μm	60.3 ± 0.1	40.5	2.9542 (13.9–90.5)
